# Sick building syndrome and indoor air quality in Malaysian bank offices: A cross-sectional analysis

**DOI:** 10.1016/j.dialog.2025.100249

**Published:** 2025-10-10

**Authors:** Azli Abd Razak, Hamidi Saidin, Rafael Buralli, Leonel Cordoba, Tengku Nilam Baizura Tengku Ibrahim, Siti Nurshahida Nazli

**Affiliations:** a School of Mechanical Engineering, College of Engineering, Universiti Teknologi MARA, 40450 Shah Alam, Selangor, Malaysia; b Department of Occupational Safety and Health, Ministry of Human Resources Malaysia, Presint 1, Putrajaya 62000, Wilayah Persekutuan Putrajaya, Malaysia; c Department of Policy, Management and Health, School of Public Health, University of Sao Paulo, SP, Brazil; d Geography School, National University, Costa Rica; e Department of Environmental Health, Faculty of Health Sciences, Universiti Teknologi MARA Cawangan Pulau Pinang, Kampus Bertam, 13200 Kepala Batas, Pulau Pinang, Malaysia; f The University of Queensland, Child Health Research Centre, South Brisbane, QLD, Australia

**Keywords:** Indoor air quality, Sick building syndrome, Ventilation, PM2.5, Carbon dioxide, Bank offices

## Abstract

Sick building syndrome (SBS) encompasses a range of non-specific symptoms experienced by occupants, commonly associated with poor indoor air quality (IAQ). This study investigated the associations between IAQ parameters and SBS symptoms among 124 office workers across six bank offices in Malaysia. Indoor PM2.5, PM10, CO2, TVOC, formaldehyde, temperature and relative humidity were measured using validated instruments, while SBS symptoms were assessed using standardized questionnaire adapted from the Industry Code of Practice of Indoor Air Quality (ICOPIAQ) 2010. Findings revealed that banks using split-unit air conditioning (AC) ventilation had higher concentration of PM2.5 and CO2, exceeding the recommended standards, compared to those using air-conditioning and mechanical ventilation (ACMV) ventilation type. The most prevalent symptoms were headache (47.6 %), cough (44.4 %), irritated and stuffy nose (38.7 %), irritation of eyes (35.5 %), fatigue (25 %), dizziness (25 %), feeling heavy-headed (24.2 %), and skin rash or itchiness (24.2 %). Among these, dizziness was significantly associated with PM2.5, PM10, CO2, and formaldehyde levels. These findings highlight the need for improved ventilation and IAQ management in bank offices. Public policies and interventions at organizational level are essential to mitigate SBS risks and safeguard worker health.

## Highlights

• This study assessed indoor air quality (IAQ) and sick building syndrome (SBS) symptoms among 124 workers in six Malaysian bank offices.
• Concentrations of PM2.5 and CO2 were higher in offices using split-unit air conditioning compared with those employing air-conditioning and mechanical ventilation systems (ACMV).
• Dizziness was the only SBS symptom significantly associated with multiple pollutants including PM2.5, PM10, CO2, and formaldehyde.
• SBS symptoms were more prevalent in buildings with poor ventilation and low air movement.
• Findings highlight the need for improved IAQ management and building ventilation strategies in bank office environments.

## 1 Introduction

Globally, individuals are spending most of their time indoors [1]. Together with thermic comfort, pollutants generated indoors and those penetrated from outdoor sources expose the occupants to significant health impacts. Indoor air quality (IAQ) describes how air inside a building can affect a person's health, comfort, and ability to work [2]. Poor IAQ has been associated with several health issues such as respiratory problems, discomfort, and nonspecific symptoms known as Sick Building Syndrome (SBS) symptoms including headaches, fatigue, eye irritation, skin dryness, throat discomfort, dizziness, and cognitive difficulties which tend to subside upon leaving the indoor environment [[3], [4], [5], [6], [7]]. According to the World Health Organization (WHO), the occupants are considered having SBS that can be associated with the IAQ or work climate if more than 20 % of individuals reported related symptoms [8].

Many studies have been conducted to assess indoor air quality and SBS symptoms towards the occupants in various type of buildings. These include hospitals, schools, homes, offices, childcare centres, small and medium industries, and business or commercial buildings [4,5,[9], [10], [11], [12], [13], [14], [15], [16], [17], [18]]. Although commercial building has been the most studied building in relation to SBS symptoms, the prevalence of SBS symptoms was relatively higher in office buildings than other different types of buildings [15,19]. Factors such as improper maintenance of centralized air-conditioning systems, poor air exchange rates and filtration systems cause accumulation of indoor air pollutants which contribute significantly to these findings. [11,16,20].

The presence of indoor air pollutants such as volatile organic compounds (VOCs), particulate matter (PM), elevated carbon dioxide (CO_2_) level, and inadequate ventilation have been identified as major contributors to SBS symptoms in office buildings and could be indicative of a risky working environment [19,[21], [22], [23]]. Other exacerbating factors include the presence of mold, high humidity levels and temperature, and exposure to traffic emissions that exacerbate the risk of SBS [5,6,11,16,23]. Due to less control over an indoor environment, there has been an increase in incidence of reported health problems [2]. In Malaysia, most studies have focused on IAQ parameters, while SBS symptoms have been less extensively studied [5].

This study assessed IAQ in Malaysian bank offices and examined the association with SBS symptoms among workers. By identifying key IAQ factors influencing SBS symptoms, this research seeks to contribute to improve workplace environment and provide data which can inform policy for maintaining optimal indoor air standards in bank offices. Assessing IAQ in bank buildings is crucial because workers are exposed in long hours in enclosed spaces, increasing the risk of health impacts from poor air quality, along with other occupational hazards. Furthermore, no studies on IAQ have been made in Malaysian banking sectors. Therefore, it is essential to determine whether bank workers are exposed to indoor pollutants that could pose potential health risks. This study seeks to contribute with comprehensive information regarding IAQ in bank buildings through diverse parameters, and SBS among bank workers from three urban areas in Malaysia.

## 2 Methodology

### 2.1 Study area and participants

This cross-sectional study was conducted in six bank offices located in Kuala Lumpur, Selangor, and Putrajaya, Malaysia. All bank offices were selected within the Klang Valley to ensure consistency in weather conditions, thus minimizing errors due to variations in temperature and humidity. Klang Valley is also known as a highly urbanized and densely populated region in Malaysia. The selected banks are also housed within the same building management and exhibit similar design and operational characteristics as shown in Table 1. All study locations were located in urban area closed to heavy traffic. They were located in Jalan Rakyat Brickfield, Kuala Lumpur, Jalan Tangsi, Kuala Lumpur, Jalan Bukit Bandar Kajang, Selangor, Seksyen 9, Shah Alam, Selangor, Subang Jaya, Selangor, and Precint 8, Putrajaya. Two bank offices were using air-conditioning and mechanical ventilation system (ACMV) and four were using split unit air-conditioning system (AC).

**Table 1:** Characteristics of the selected bank offices.

Parameter	Bank 1	Bank 2	Bank 3	Bank 4	Bank 5	Bank 6
Location	Brickfield, KL	Jalan Tangsi, KL	Kajang	Seksyen 9, Shah Alam	USJ, Subang Jaya	Putrajaya
Type of building	Ground floor high rise	Ground floor high rise	2 storey commercial	2 storey commercial	2 storey commercial	3 storey commercial
Year of construction	2014	1982	2008	1996	1996	2015
Ventilation type	ACMV	ACMV	AC	AC	AC	AC
Operation Division volume [m^2^]	231.2	348.2	114.9	111.5	110.0	127.0
Occupant (min-max)	9–18	8–16	10–26	10–34	6–18	4–22
Door area [m^2^]	4.1	4.1	4.1	4.1	4.1	4.1
Floor type [Operation Division]	Tile & Carpet	Tile	Tile	Tile	Tile	Tile & Carpet

**Fig. 1:**
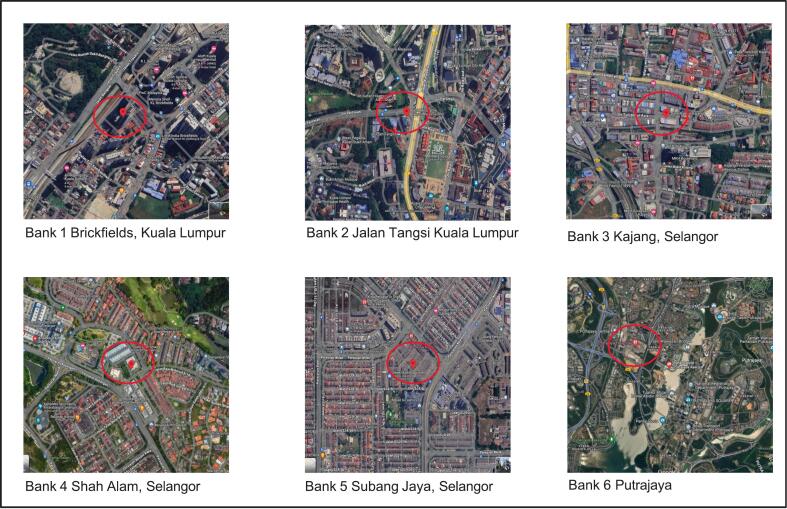
Maps of study locations. Red circles represent the locations of the bank offices. (For interpretation of the references to colour in this figure legend, the reader is referred to the web version of this article.)

Fig. 1 illustrates the map of the study locations. All eligible administrative staff who had worked at the selected banks for at least six months and were willing to participate in the study were invited. There were 21, 28, 18, 19, 19 and 19 respondents in Bank 1 until Bank 6, respectively. A total of 124 administrative employees from the six selected bank offices participated in the study through complete enumeration.

### 2.2 Data collection

#### 2.2.1 IAQ Measurements

IAQ parameters concentration were measured at operation division and vault room. These two areas were chosen to represent the main operations of a bank office. Twelve sampling locations were selected within these areas and average readings between these areas were calculated to present the concentrations of IAQ parameters. Chemical and physical parameters including PM_2.5_, PM_10_, CO_2_, CO, TVOC, formaldehyde, temperature, relative humidity and air movement were measured within working hours and compared to the related IAQ standards. The air sampling procedure and sampling locations for assessing IAQ parameters adhered to the guidelines outlined by ICOP-IAQ (2010). Sampling was conducted throughout the entirety of bank operations to accurately depict IAQ conditions within the office environment during the work shift. The specific number of measurements conducted refers to the recommended minimum number of sampling points in Table 2.

**Table 2:** Recommended minimum number of sampling points (DOSH, 2010).

Total floor area (m^2^)	Minimum number of sampling points
< 3000	1 per 500 m^2^
3000 - < 5000	8
5000 - < 10,000	12
10,000 - < 15,000	15
15,000 - < 20,000	18
20,000 - < 30,000	21
> 30,000	1 per 1200 m^2^

Diverse instruments were used for measuring CO and CO2 levels, including IAQ Monitor, Fluke 975 AirMeter and QUEST AQ5000 Air Quality Monitor, while PpbRAE 3000 Organic Vapor Monitor from RAE USA was used for detecting TVOC. DUSTTRAK DRX Aerosol Monitor 8534 from TSI US was utilized for quantifying particulate matter, specifically PM10 and PM2.5. Formaldemeter htV was used to measure formaldehyde concentrations, while VelociCalc Multi-Function Ventilation Meter from TSI USA was used to measure air velocity, temperature and humidity. To ensure the accuracy and reliability of the collected data, stringent quality assurance and quality control measures were implemented throughout the study. Prior to conducting any measurements, pre-checks and post-checks of the measurement equipment were performed to verify their proper functioning. All equipment employed in the study was calibrated by authorized calibration bodies and held valid certificates of calibration, thereby ensuring the traceability and accuracy of the measurements. These measures aim to minimize measurement errors and ensure the validity of the collected data.

#### 2.2.2 Questionnaire survey

A validated questionnaire adopted from the Industry Code of Practice of Indoor Air Quality (ICOP-IAQ) in 2010 was used to assess SBS symptoms among participants. The survey employed complete enumeration, encompassing participation from all 124 employees, thus ensuring that the entire population was sampled to accurately reflect the survey results. There are two sections in the questionnaire: i) covering participant's background information and environmental conditions of the work area, and ii) on present and past symptoms of SBS experienced by the respondents.

### 2.3 Data analysis

Data analysis was performed in RStudio v4.3.1. Descriptive statistics and boxplot were used to depict IAQ measurements and compare them to the relevant standards (ICOP-IAQ, ASHRAE, WHO) as shown in Table 3. The prevalence of SBS symptoms among respondents across study locations were also presented. Correlation analysis was conducted using Pearson's correlation coefficients to assess bivariate relationships between air pollutant concentrations and SBS symptom prevalence. Since all variables were continuous and the sample size was moderate, Pearson was selected as the method. Multiple linear regression models were then applied to evaluate the effect of individual pollutants on specific symptoms, using symptom-specific prevalence as the dependent variable and pollutant levels (PM2.5, PM10, CO₂, CO, TVOC, formaldehyde, temperature, relative humidity, and air movement) as predictors.

**Table 3:** Standards of indoor air quality parameters.

Parameter	ICOP-IAQ	ASHRAE	WHO
Relative Humidity	40–70 %	30–65 %	
Temperature	23–26 °C	22–26 °C	
Carbon dioxide	1000 ppm	700 ppm	
Carbon monoxide	10 ppm	9 ppm	
PM10	0.15 mg/m3		
PM2.5			0.025 mg/m3
TVOC	3000 ppb		
Formaldehyde	0.1 ppm		
Air movement	0.15–0.5 m/s		

## 3 Result

### 3.1 Demographic characteristics

A total of 124 administrative bank employees participated in the study, with a 100 % response rate. The mean age of respondents was 37.4 (range 20–59) years old. About 57.3 % of respondents were male while 42.7 % were female. The median employment time was 9.4 years. About 29 % of the respondents were smokers.

### 3.2 Indoor air quality measurements

**Fig. 2:**
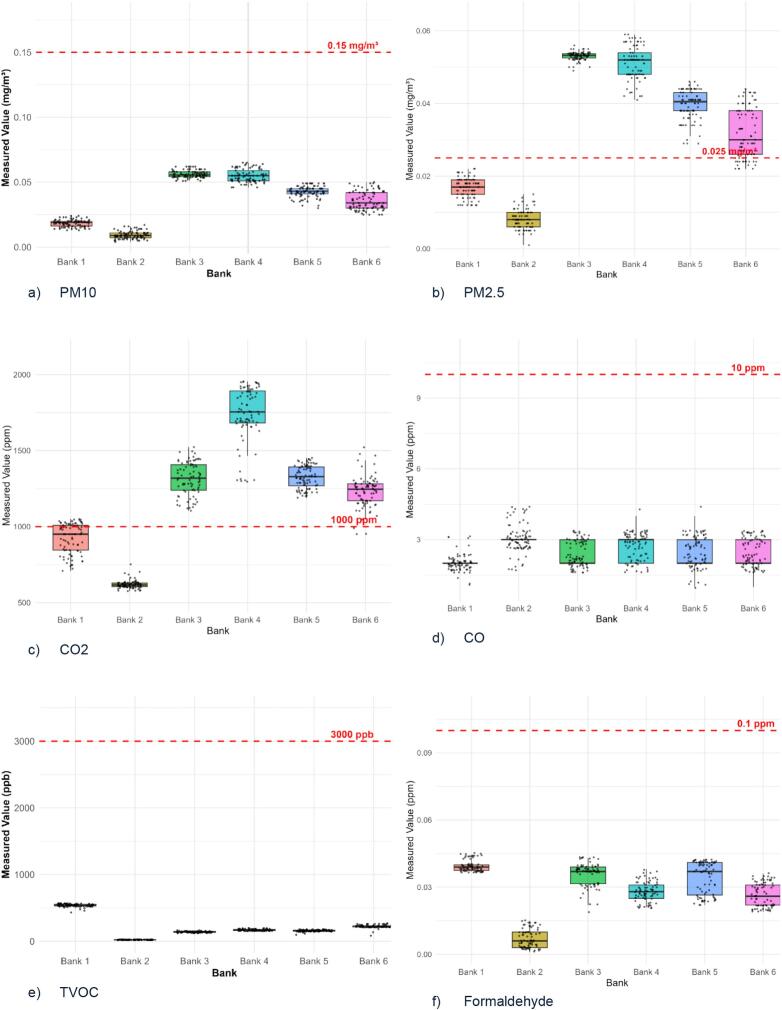

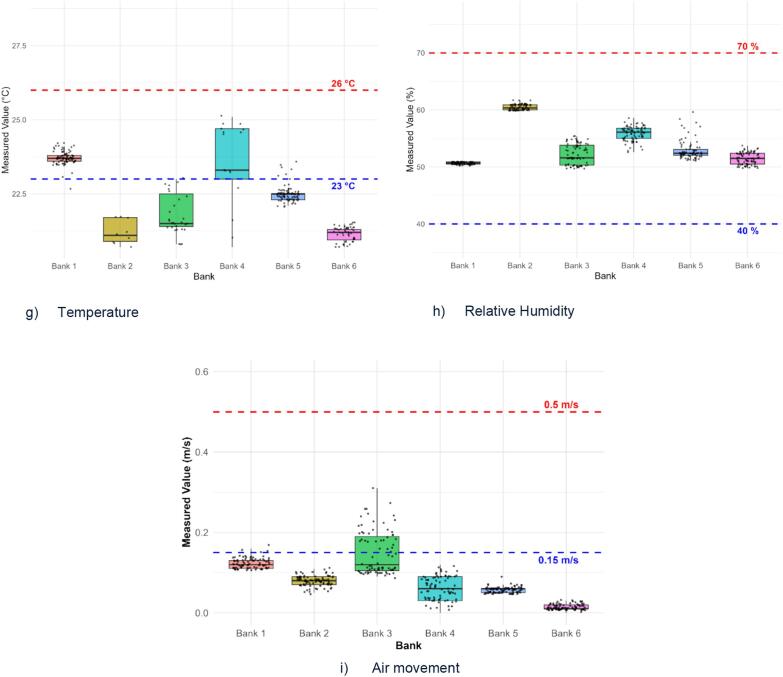
Boxplots showing the concentration levels distribution of (a) PM10, (b) PM2.5, (c) CO2, (d) CO, (e) TVOC, (f) formaldehyde, (g) temperature, (h) relative humidity, and (i) air movement across six bank offices.

Fig. 2 depicts the distribution of IAQ parameters concentrations across study locations. PM_10_ levels across the study locations were far below the ICOP-IAQ standard of 0.15 mg/m^3^, with the lowest concentrations observed in Bank 1 and Bank 2, which used ACMV ventilation type (Fig. 2a). PM2.5 concentrations exceeded the WHO standard of 0.025 mg/m^3^ in most banks, including Banks 3, 4, 5 and 6 (AC ventilation type), while Bank 1 and 2 remained below the limit (Fig. 2b). Concentration of CO2 at all study locations also exceeded the ICOP-IAQ limit of <1000 ppm, except in Bank 1 and Bank 2 (Fig. 2c). However, no locations exceeded the standard limits for CO, TVOC, and formaldehyde (Fig. 2d-1f). Temperature levels were only seen within the recommended range of 23 °C to 26 °C in Bank 1 and Bank 4, while other locations recorded below 23 °C. Relative humidity remained within 40 % - 70 % range at all study locations (Fig. 2h). Air movement was below the ICOP-IAQ range of 0.15–0.5 m/s in all locations. Highest air movement was recorded at Bank 3 at 0.31 m/s, while other locations had minimal air movement.

### 3.3 Prevalence of SBS symptoms

**Fig. 3:**
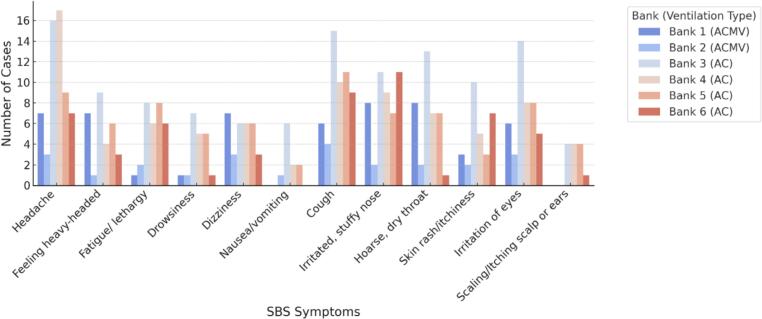
Prevalence of SBS Symptoms among respondents by bank and ventilation type.

Fig. 3 depicted the prevalence of SBS symptoms reported by participants across six bank locations with two different types of ventilation, in which Bank 1 and Bank 2 employed ACMW, while Bank 3, Bank 4, Bank 5 and Bank 6 employed AC ventilation type. Headache was the most frequent reported symptom (47.6 %), followed by cough (44.4 %), irritated and stuffy nose (38.7 %), irritation of eyes (35.5 %), fatigue (25 %), dizziness (25 %), feeling heavy-headed (24.2 %), and skin rash or itchiness (24.2 %). Other symptoms like drowsiness, scaling/itching scalp or ears, and nausea were reported by less than 20 % of participants. From the figure, participants in Bank 4 reported the highest prevalence of headaches (89.5 %), followed by Bank 3 (88.9 %), which was higher than other locations. Bank 3 also recorded highest prevalence of cough (83.3 %), hoarse, dry throat (72.2 %), skin rash/itchiness (55.6 %), and irritation of eyes (77.8 %). The result revealed different variations in the prevalence of symptoms, which were more pronounced in AC-ventilated banks than ACMV-ventilated banks.

### 3.4 Correlation between IAQ parameters and SBS symptoms

Normality of the IAQ parameters and SBS symptom data was assessed using the Shapiro–Wilk test. Most variables showed no significant deviation from normality (*p* > 0.05), justifying the use of Pearson correlation. However, variables such as *Dizziness* and *Scaling/Itching Scalp or Ears* did not meet the assumption of normality (*p* < 0.05); thus, Spearman's rank correlation was applied for associations involving these symptoms.

**Fig. 4:**
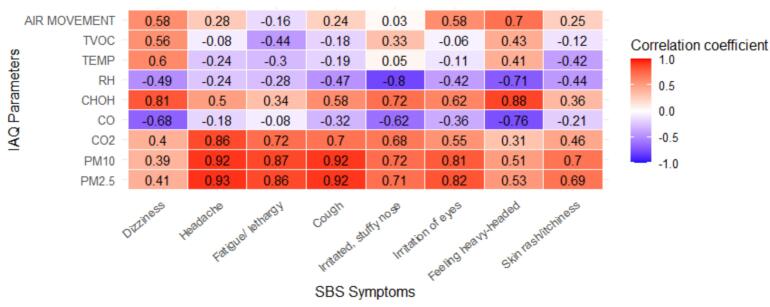
Correlation heatmap between indoor air quality (IAQ) parameters and SBS symptoms among bank office workers. Red indicates positive correlations; blue indicates negative correlations. Abbreviations: TVOC = Total Volatile Organic Compounds; TEMP = Temperature; RH = Relative Humidity; HCHO = Formaldehyde); CO = Carbon Monoxide; CO₂ = Carbon Dioxide; PM10 = Particulate Matter ≤10 μm; PM2.5 = Particulate Matter ≤2.5 μm. SBS symptoms: Dizziness, headache, fatigue/lethargy, cough, irritated/stuffy nose, irritation of eyes, feeling heavy-headed, and skin rash/itchiness. (For interpretation of the references to colour in this figure legend, the reader is referred to the web version of this article.)

Correlation matrix in Fig. 4 shows the relationship between measured IAQ parameters with SBS symptoms. We only included the symptoms with more than 20 % complaints in the analysis. Correlation coefficient in red colour (*r* > 0.7) indicates strong positive correlations while purple colour (*r* < −0.5) indicates strong negative correlations. The results show that increment in several IAQ parameters were significantly associated with SBS symptoms among participants. PM2.5 showed a strong positive correlation with headache, cough, fatigue/lethargy, and irritation of eyes. Similar correlations were seen between PM10 and these symptoms. CO2 was positively associated with headache while formaldehyde was positively associated with dizziness and feeling heavy-headed. Air movement was only associated with feeling heavy-headed. Conversely, relative humidity was strongly negatively associated with irritated, stuffy nose and feeling heavy-headed, while CO was strongly associated with dizziness, feeling heavy-headed and irritated, stuffy nose.

Pearson correlation analysis further supported the above finding. Table 4 presents the Pearson correlation coefficients (r) for each measured IAQ parameters. We observed that PM2.5 had the strongest positive correlation with SBS symptoms prevalence, followed closely by PM10, and CO₂. In contrast, relative humidity, and CO and air movement showed weak correlations. Temperature and TVOC also showed very weak correlations with the prevalence symptoms. The results showed particulate matter including PM2.5 and PM10, and elevated CO2 concentrations are significant contributors to SBS symptoms among respondents in the studied bank offices.

**Table 4:** Pearson correlation coefficients between IAQ parameters and overall SBS symptom prevalence.

IAQ parameter	Correlation with SBS Symptom Prevalence (r)
Air Movement	0.27
Formaldehyde	0.41
CO	−0.24
CO2	0.44
PM10	0.56
PM2.5	0.57
RH	−0.29
Temp	−0.07
TVOC	−0.03

Positive values indicate a direct relationship while negative values suggest an inverse relationship.

### 3.5 Multiple linear regression analysis of pollutant effects on SBS symptoms

Multiple linear regression was conducted in which all symptoms were assessed in a model with all IAQ parameters. The result revealed that dizziness was the only SBS symptom significantly correlated with multiple indoor air pollutants, including PM2.5, PM10, CO2, and formaldehyde (Table 5). While other symptoms showed elevated regression coefficients, their correlations were not statistically significant. Relative humidity (RH) was automatically excluded due to multicollinearity, particularly with formaldehyde and temperature. As air movement was not highly correlated with other IAQ parameters, it was not retained in the final model, likely due to limited variance or lack of predictive power.

**Table 5:** Multiple linear regression analysis showing significant predictors of dizziness.

Symptoms	Term	Estimate	Std.Error	Statistic	*p*-value
Dizziness	PM2.5	1664.27	11.52	144.453	0.004
	PM10	−1669.29	11.46	−145.64	0.004
	CO2	0.01	5.38e-05	93.69	0.007
	Formaldehyde	93.99	0.70	133.49	0.005

## 4 Discussion

This study evaluated indoor air quality (IAQ) parameters including PM2.5, PM10, CO, CO_2_, TVOC, formaldehyde, air movement, temperature and relative humidity in six bank offices and assessed their association with SBS among workers. IAQ and the effects to SBS symptoms studies have been conducted extensively in recent years, yet lack of study was found in bank offices, where workers spend long working hours in enclosed spaces.

Our findings revealed a high prevalence of SBS symptoms among bank workers, particularly headache, cough, irritated/stuffy nose, eye irritation, dizziness, and fatigue. These symptoms were identified to be influenced by the elevated levels of PM2.5, PM10, and CO2 in the office buildings. Dizziness was the only symptom significantly associated with multiple indoor air pollutants, including PM2.5, PM10, CO2, and formaldehyde as key contributors. This finding aligns with previous studies that have identified particulate matter and CO2 as significant parameters affecting IAQ and major contributors to SBS complaints [15]. While other SBS symptoms may still be influenced by poor IAQ, their associations did not reach statistical significance in our model, possibly due to individual variability or confounding factors. These results underscore the need for further studies to explore why dizziness appears more sensitive to pollutant exposure and whether it could serve as an early indicator of poor indoor air quality.

The elevated levels of indoor CO_2_ concentrations have exceeded the ICOP-IAQ 2010 threshold in all AC ventilation type bank offices, indicating poor ventilation and high occupancy levels. Elevated CO2 concentrations have been associated with discomfort, cognitive impairment, and SBS manifestations, such as headache and dizziness [16]. Our findings suggest that human activities and poor ventilation in bank offices generated high CO2 levels in the indoor environment as supported by other studies [5,[15], [16], [17],^21^]. Additionally, CO levels were also measured higher in AC ventilation type buildings, indicating that AC buildings near high-traffic areas could lead to infiltration from outdoor sources, causing dizziness, headache, and nausea (Subri et al., 2024; Mansor et al.,2024; Thach et al.,2019). However, the observed negative association between CO and SBS symptoms was inconsistent with toxicological knowledge, suggesting that our result was likely influenced by non-measured confounding factors or indirect relationships which warrant further analysis.

Thermal comfort conditions were suboptimal in most bank offices, with indoor temperature consistently below the recommended level of 23 °C to 26 °C. Despite this, temperature was not associated with any SBS symptom prevalence in our analysis, which is also supported with prior study suggesting that lower temperature may reduce the risk to SBS symptoms [19]. Conversely, higher temperature was associated with headache, feeling heavy-headache, and skin rash itchiness and increased nasal symptoms among administrative office workers [5,19]. Relative humidity levels were within acceptable levels, though was negatively associated with irritated, stuffy nose and feeling heavy-headed. These finding suggest that low relative humidity may exacerbate SBS symptoms due to mucosal dryness [11].

Air movement in all bank offices had low air movement below standards, indicating poor ventilation effectiveness and limited fresh air intake, which also supports the high indoor CO_2_ levels [15,17]. The insufficient air movement can cause stagnation zones that exacerbate exposure to pollutants, particularly in offices located near busy roadways [11,21]. Poor air movement was associated with feeling heavy-headed, similar to finding by Mansor et al. (2024). We did not observed association of air movement with eyes, nose, and throat irritation, headache and fatigue as found by other study [15]. Although formaldehyde levels were within standard limits, they were still associated with dizziness and feeling heavy-headed. Evidence suggests that even in low- exposure settings, chronic exposure to formaldehyde can cause neurological and mucosal effects [20,21]. This hazardous pollutant is emitted by some building materials, furniture and consumer goods, which pose a concern in closed office environments [15].

These findings highlight the need for improved ventilation design and maintenance in the banking sector, where employees spend long hours in indoors. Strengthening occupational health policies and targeted IAQ interventions are essential to safeguard worker well-being and productivity.

There were few limitations identified in this study. First, the relatively small number of bank offices assessed in the study limits the generalizability of the findings. Second, the cross-sectional design prevents causal inference between IAQ parameters and SBS symptoms. Third, SBS symptoms were self-reported, which may introduce recall or reporting bias although the questionnaire was validated to ensure reliability. Additionally, some statistically significant results may have occurred by chance, and the observed associations between IAQ parameters and SBS symptoms require confirmation in future investigations. We were unable to assess the impact of different ventilation systems due to the small sample size and could not adjust for individual habits or organizational factors that may influence symptom reporting. Finally, as the study was conducted over a short period, longitudinal research with larger samples is recommended to strengthen causal understanding.

## 5 Conclusion

This study highlights a clear association between elevated levels of PM2.5, PM10, and CO2 with SBS symptoms among bank office workers in Malaysia. Symptoms such as headaches, cough, irritated and stuffy nose, irritation of eyes, dizziness, feeling heavy-headed and skin rash or itchiness were particularly prevalent in offices using AC ventilation type than ACMV ventilation type. Among the reported symptoms, dizziness showed the most consistent associations with measured IAQ parameters. However, this observation should be interpreted cautiously, as the study design does not allow causal inference. Further research with larger samples and longitudinal approaches is required before dizziness can be considered a potential early marker of IAQ problems. The findings also emphasize the urgent need for improved ventilation design, regular maintenance of HVAC systems, and implementation of IAQ monitoring protocols in the banking sector. Future research should employ longitudinal designs with larger sample size to better understand causal relationships and explore the effectiveness of intervention strategies. It is necessary to implement public policies and measures, prioritizing indoor air quality to ensure healthy and productive work environments.

## CRediT authorship contribution statement

**Azli Abd Razak:** Writing – review & editing, Validation, Supervision, Methodology, Conceptualization. **Hamidi Saidin:** Writing – original draft, Methodology, Investigation, Conceptualization. **Rafael Buralli:** Writing – review & editing, Visualization. **Leonel Cordoba:** Writing – review & editing, Visualization. **Tengku Nilam Baizura Tengku Ibrahim:** Writing – review & editing, Visualization. **Siti Nurshahida Nazli:** Writing – review & editing, Writing – original draft, Visualization, Validation, Methodology, Formal analysis, Data curation, Conceptualization.

## Funding sources

This research did not receive any specific grant from funding agencies in the public, commercial, or not-for-profit sectors.

## Declaration of competing interest

The authors declare that they have no known competing financial interests or personal relationships that could have appeared to influence the work reported in this paper.
